# Is income or employment a stronger predictor of smoking than education in economically less developed countries? A cross-sectional study in Hungary

**DOI:** 10.1186/1471-2458-11-97

**Published:** 2011-02-13

**Authors:** Mall Leinsalu, Csilla Kaposvári, Anton E Kunst

**Affiliations:** 1 Stockholm Centre on Health of Societies in Transition (SCOHOST), Södertörn University, Huddinge, Sweden; 2 Centre for Health Equity Studies (CHESS), Stockholm University/Karolinska Institute, Stockholm, Sweden; 3 Health Monitor Nonprofit Public Benefit Ltd., Budapest, Hungary; 4 Department of Public Health, Erasmus University Medical Centre, Rotterdam, The Netherlands; 5 Department of Public Health, Amsterdam Medical Centre, University of Amsterdam, Amsterdam, The Netherlands

## Abstract

**Background:**

In developed European countries in the last phase of the smoking epidemic, education is a stronger predictor of smoking than income or employment. We examine whether this also applies in economically less developed countries.

**Methods:**

Data from 7218 respondents in the 25-64 age group came from two National Health Interview Surveys conducted in 2000 and 2003 in Hungary. Independent effects of educational level, income and employment status were studied in relation to smoking prevalence, initiation and continuation for all age groups combined and separately for 25-34, 35-49 and 50-64 years old. Absolute levels were evaluated by using age-standardized prevalence rates. Relative differences were assessed by means of logistic regression.

**Results:**

Education and income, but not employment, were associated with equally large differences in smoking prevalence in Hungary in the 25-64 age group. Among men, smoking initiation was related to low educational level, whereas smoking continuation was related to low income. Among women, low education and low income were associated with both high initiation and high continuation rates. Considerable differences were found between the age groups. Inverse social gradients were generally strongest in the youngest age groups. However, smoking continuation among men had the strongest association with low income for the middle-aged group.

**Conclusions:**

Patterns of inequalities in smoking in Hungary can be best understood in relation to two processes: the smoking epidemic, and the additional effects of poverty. Equity orientated tobacco control measures should target the low educated to prevent their smoking initiation, and the poor to improve their cessation rates.

## Background

Tobacco smoking is an important cause of premature mortality, and particularly so in Eastern Europe [1]. In 2000, more than one third of all male deaths in the age group 35-69 were attributed to smoking in the countries of Central and Eastern Europe [2]. Smoking also has a large impact on social inequalities in mortality [3,4]. According to a recent study of 16 European countries, smoking-related conditions accounted for 21% of the educational inequalities in all-cause mortality among men and 6% of those among women [5].

Socioeconomic inequalities in smoking are largely determined by the progression of the smoking epidemic [6]. According to this model, the smoking prevalence among men peaks when cigarette prices become affordable for all socioeconomic groups and starts declining when higher socioeconomic groups first quit smoking. As a result, the social gradient in smoking reverses. In women, these patterns usually lag 10-20 years behind those of men [7]. Despite the large burden of smoking-related mortality, the history of the smoking epidemic is much less documented for Eastern Europe [8-12]. Most of these studies reveal a negative association between smoking prevalence and socioeconomic status among men. Among women this association is less clear, although in the younger age groups it also tends to be negative for women [13].

Educational level, occupation and income have all been used as indicators of individuals' socioeconomic status, each of them representing their own causal pathways in explaining inequalities in health [14]. Lower levels of education, manual occupations and material disadvantage have also been related to higher rates of smoking [12,15,16]. For European populations in the last phase of the smoking epidemic, a strong association between educational level and smoking prevalence has been recorded for both men and women [13,16]. In multivariate analyses, education has been found to be a more important predictor of smoking than income within these countries [16]. In Central and Eastern Europe, where the overall living standards are still much lower when compared to Western Europe, low income levels are more likely to be linked to both absolute poverty and economic insecurity. In such circumstances income or employment related differences in smoking might be larger than those related to educational level.

To test this hypothesis, we conducted a case study in Hungary, a former communist country in Central Europe with 10 million inhabitants. In 2004, Hungary's per capita gross domestic product (in purchasing power standards) was 56% of that of the 15 "old" EU member states [17]. In around 2000, men in Hungary had the highest smoking attributable mortality among all men in Europe in the 35-69 age group (42% of all deaths), while Hungarian women ranked second after Denmark [2]. Compared to the mid-1990 s, the smoking prevalence has fallen in adult men but not in women. The social context of adult tobacco consumption, however, has remained little explored.

Our main research question is whether in a country like Hungary, inequalities in smoking follow the patterns predicted by the smoking epidemic model, i.e. those with higher education being the forerunners of the trends in smoking. Alternatively, income and other indicators of adult socioeconomic position like employment may be more important predictors of smoking compared to educational level. By using multivariate analysis, we assess the independent effect of each of these indicators on smoking prevalence. We extend our analysis to smoking initiation and smoking continuation which are the component parts of smoking prevalence. Smoking initiation and cessation have shown different patterns of social inequalities [12] and thus, may help us to better assess the effects of education as well as other socioeconomic factors on smoking. Moreover, as smoking initiation and cessation relate to the transition between different stages in tobacco consumption [18] they can be better targeted by tobacco control policies.

## Methods

### Study population

We pooled the datasets from the two National Health Interview Surveys. The surveys were conducted as face-to-face interviews in 2000 and 2003 on the same methodological basis [19]. The Central Data Processing, Registration and Election Office's registry which includes all Hungarian citizens aged 18 years and older was utilized as a sample frame. A stratified two-stage sampling procedure was used. In the first stage, 440 settlements (447 in 2003) were selected by using inclusion probabilities proportional to the settlement size, except for the large settlements which had an inclusion probability of 1. Thereafter, 7000 individuals were selected without replacement by using simple random sampling. The response rate was 78.6% (N = 5503) in 2000 and 72.4% (N = 5072) in 2003. The non-location of respondents was the most frequent reason for non-response (12.5% of the initial sample in 2000 and 9.8% in 2003) followed by refusal to participate (7.5% and 8.2%). Both surveys were representative of the Hungarian population in terms of age, gender and place of residence [20,21]. We limited our study to the 25-64 age group, thus including 3322 men and 3896 women. The item non-response was below 1% for all study variables except for income where 14.8% of the answers were missing for men and 13.1% for women. All cases with missing answers were additionally excluded from the analysis.

### Dependent variables

Information on tobacco consumption was obtained from several questions on the respondent's smoking habits. The answers were reclassified in a comparable way into three variables. *Current smoking *measures the proportion of daily smokers and occasional smokers among all respondents.

*Ever initiating smoking *(hereafter *smoking initiation*) measures the proportion of current smokers and ex-smokers among all respondents.

*Continuation of smoking *measures the proportion of daily and occasional smokers among ever smokers.

### Independent variables

*Educational level *was determined according to diploma level and respondents were classified into four categories corresponding to the International Standard Classification of Education: higher education (ISCED categories 5-6), upper secondary education (3-4), lower secondary education (2), and no or only primary education (0-1).

For *employment status *economically active respondents were divided into three groups: non-manual employees, manual workers, and self-employed. Farmers and farm labourers were classified as manual workers. The *non-employed *group combined all economically inactive individuals, thus including the unemployed, students, housewives, work disabled or retired individuals.

To measure the *household income *respondents were asked to estimate the total monthly net income of the people in their household. Total income was then divided by the square root of the household size to get the household equivalent income which was thereafter divided into quintiles.

The association with tobacco consumption was also assessed for two sociodemographic variables. For *marital status *respondents were classified into three groups: married or living with a partner, never married, and divorced, separated or widowed. *Place of residence *distinguished between respondents living in urban or rural areas.

### Statistical methods

We calculated age-standardized prevalence rates to evaluate absolute differences. Direct standardization with 5-year age groups was used with the total Hungarian population as a standard. Relative differences were assessed by means of logistic regression using the SPSS 16.0 statistical package and results are presented as odds ratios with 95% confidence intervals. The effect of each independent variable was measured in bivariate analysis adjusting only for age (Model 1), and in multivariate analysis adjusting mutually for age and all socioeconomic and sociodemographic variables (Model 2). Age was included into all models in 5-year groups and all analyses were performed separately for men and women. The association of socioeconomic variables with smoking initiation and smoking continuation was additionally studied in the 25-34, 35-49 and 50-64 age groups. To assess the possible impact of multicollinearity among the variables in the multivariate analysis, we calculated the variance inflation factor (VIF) for each variable in the regression model. The largest detected VIF value was 1.5, indicating that our results are not affected by multicollinearity.

### Ethical considerations

Both surveys have been approved by the Ethical Committee of the Hungarian National Scientific Council on Health. The data we used are anonymous and publicly available on the condition that official request is made to the data holder, i.e. the Hungarian National Centre for Epidemiology.

## Results

According to our study, 69% of men and 48% of women in the 25-64 age group had ever initiated smoking. 35% of male and 33% of female ever smokers had quit by the time of the surveys. The prevalence rate of current smoking was 44% among men and 33% among women.

The results for current smoking are presented in Table 1. Smoking was more prevalent among men and women who were low educated or who had a low income (especially those on the lowest income level). After adjustment for other socioeconomic and sociodemographic variables, men and women with the lowest educational level and with the lowest income level still had about two times higher odds of being current smokers when compared to those with the highest educational level or highest income. Being a manual worker or non-employed was associated with current smoking in the bivariate model but not in the multivariate model.

**Table 1:** Age-standardized prevalence rates (PR) and odds ratios (OR) with 95% confidence intervals (CI) for current smoking in the 25-64 age group in Hungary, 2000-2003.

		Men						Women					
			
				Model 1		Model 2				Model 1		Model 2	
									
		N	PR (%)	OR	95% CI	OR	95% CI	N	PR (%)	OR	95% CI	OR	95% CI
**Total**		3322	44.3	-		-		3896	32.6	-		-	
													
**Marital status**													
	Married	2341	41.3	1.00		1.00		2585	29.5	1.00		1.00	
	Never married	629	48.9	1.23	(1.02, 1.48)	1.13	(0.91, 1.39)	432	40.6	1.17	(0.94, 1.46)	1.13	(0.88, 1.44)
	Divorced/separated/widowed	347	57.4	2.04	(1.62, 2.57)	1.74	(1.35, 2.26)	878	41.7	1.54	(1.30, 1.82)	1.37	(1.14, 1.65)
													
**Urban/rural residence**													
	Urban	2009	41.6	1.00		1.00		2486	32.8	1.00		1.00	
	Rural	1313	48.3	1.34	(1.16, 1.54)	1.05	(0.89, 1.23)	1410	31.8	0.93	(0.81, 1.07)	0.79	(0.67, 0.93)
													
**Education**													
	Higher	512	28.0	1.00		1.00		611	23.0	1.00		1.00	
	Upper secondary	826	37.7	1.54	(1.21, 1.96)	1.38	(1.03, 1.85)	1311	28.7	1.37	(1.10, 1.72)	1.33	(1.02, 1.74)
	Lower secondary	1289	48.3	2.40	(1.92, 3.00)	1.82	(1.33, 2.49)	752	38.3	2.20	(1.73, 2.80)	1.91	(1.39, 2.64)
	Primary or less	686	57.9	3.21	(2.50, 4.11)	2.08	(1.48, 2.93)	1205	42.4	2.16	(1.72, 2.72)	1.95	(1.42, 2.69)
													
**Employment**													
	Non-manual employees	589	34.7	1.00		1.00		1014	27.7	1.00		1.00	
	Manual workers	1269	47.9	1.83	(1.50, 2.25)	1.07	(0.81, 1.41)	866	37.9	1.88	(1.55, 2.28)	1.27	(0.98, 1.65)
	Self-employed	427	34.5	1.05	(0.81, 1.36)	0.86	(0.62, 1.18)	210	31.1	1.35	(0.98, 1.86)	1.18	(0.79, 1.76)
	Non-employed	985	52.2	1.96	(1.57, 2.45)	0.93	(0.68, 1.26)	1764	35.7	1.29	(1.08, 1.54)	0.81	(0.63, 1.03)
													
**Household income**													
	1 Highest	686	36.9	1.00		1.00		705	26.3	1.00		1.00	
	2	562	39.5	1.15	(0.91, 1.45)	1.00	(0.78, 1.28)	654	25.6	0.97	(0.76, 1.24)	0.87	(0.67, 1.12)
	3	544	44.4	1.45	(1.15, 1.83)	1.16	(0.90, 1.49)	698	32.1	1.35	(1.07, 1.71)	1.13	(0.88, 1.46)
	4	464	49.6	1.78	(1.40, 2.27)	1.36	(1.04, 1.78)	657	34.0	1.35	(1.06, 1.72)	1.11	(0.84, 1.45)
	5 Lowest	576	57.8	2.51	(2.00, 3.16)	1.77	(1.35, 2.33)	673	46.6	2.42	(1.93, 3.05)	2.01	(1.53, 2.65)

Model 1 - adjusted for age.

Model 2 - adjusted for age, marital status, place of residence, education, employment status and household income.

Divorced, separated or widowed men and women were more likely to smoke compared to those who were married/cohabiting. Rural women had 21% lower odds of being smokers compared to urban women. No urban/rural differences were observed among men in the multivariate model.

Table 2 presents the results of the multivariate model for ever initiating smoking and for smoking continuation. A clear inverse educational gradient was observed for smoking initiation among men; those with the lowest educational level had 2.3 times higher odds for smoking initiation compared to the highest educated men. Neither income nor employment status were associated with smoking initiation among men in the multivariate model. Similar to men, women with less than higher education had an elevated risk to initiate smoking though the association was statistically not significant for the lowest educated. Unlike men, women in the lowest income group had 1.7 times higher odds to have started smoking compared to the highest income group. Employment status was also not associated with smoking initiation among women.

**Table 2:** Age-standardized prevalence rates (PR) and odds ratios (OR) with 95% confidence intervals (CI) for smoking initiation and continuation in the 25-64 age group in Hungary, 2000-2003.

		Men						Women					
			
		Smoking initiation			Smoking continuation			Smoking initiation			Smoking continuation		
					
			Model 2			Model 2			Model 2			Model 2	
									
		PR (%)	OR	95% CI	PR (%)	OR	95% CI	PR (%)	OR	95% CI	PR (%)	OR	95% CI
**Total**		68.9	-		65.1	-		47.7	-		67.2	-	
													
**Marital status**													
	Married	68.0	1.00		61.5	1.00		45.1	1.00		64.3	1.00	
	Never married	70.1	0.91	(0.73, 1.14)	71.3	1.40	(1.04, 1.89)	53.3	0.94	(0.74, 1.19)	75.5	1.52	(1.03, 2.24)
	Divorced/separated/widowed	75.8	1.41	(1.04, 1.91)	76.5	1.79	(1.29, 2.47)	56.2	1.29	(1.08, 1.53)	72.6	1.27	(0.97, 1.65)
													
**Urban/rural residence**													
	Urban	66.4	1.00		63.7	1.00		48.9	1.00		66.2	1.00	
	Rural	72.5	1.01	(0.84, 1.21)	67.1	1.04	(0.85, 1.28)	45.2	0.79	(0.68, 0.92)	69.1	0.90	(0.71, 1.15)
													
**Education**													
	Higher	53.2	1.00		55.2	1.00		41.9	1.00		55.1	1.00	
	Upper secondary	62.9	1.47	(1.11, 1.95)	60.7	1.04	(0.71, 1.51)	47.0	1.29	(1.02, 1.62)	60.8	1.17	(0.82, 1.66)
	Lower secondary	73.6	2.13	(1.57, 2.90)	66.2	1.14	(0.77, 1.68)	53.5	1.57	(1.18, 2.10)	69.3	1.69	(1.09, 2.63)
	Primary or less	79.8	2.32	(1.64, 3.29)	72.1	1.28	(0.83, 1.96)	52.9	1.31	(0.99, 1.74)	77.3	2.29	(1.47, 3.56)
													
**Employment**													
	Non-manual employees	59.5	1.00		59.8	1.00		48.5	1.00		59.4	1.00	
	Manual workers/farmers	72.0	1.21	(0.91, 1.60)	67.2	0.95	(0.67, 1.37)	49.8	1.20	(0.94, 1.52)	77.7	1.27	(0.87, 1.84)
	Self-employed	59.2	0.91	(0.66, 1.24)	58.3	0.81	(0.53, 1.23)	47.4	1.16	(0.80, 1.68)	60.0	1.08	(0.63, 1.87)
	Non-employed	73.7	1.14	(0.83, 1.55)	71.5	0.86	(0.58, 1.27)	50.6	0.86	(0.69, 1.07)	68.7	0.79	(0.56, 1.10)
													
**Household income**													
	1 Highest	64.2	1.00		58.9	1.00		43.9	1.00		58.6	1.00	
	2	66.8	0.91	(0.71, 1.17)	59.9	1.04	(0.76, 1.41)	42.5	0.89	(0.71, 1.12)	59.7	0.89	(0.63, 1.26)
	3	70.1	0.99	(0.76, 1.29)	64.1	1.25	(0.91, 1.71)	47.2	1.07	(0.85, 1.35)	68.7	1.13	(0.80, 1.61)
	4	72.4	1.01	(0.76, 1.35)	68.9	1.52	(1.08, 2.14)	48.0	1.03	(0.80, 1.31)	68.8	1.14	(0.78, 1.68)
	5 Lowest	77.6	1.24	(0.92, 1.68)	74.5	1.97	(1.39, 2.79)	58.5	1.72	(1.33, 2.22)	77.5	1.80	(1.20, 2.69)

Model 2 - adjusted for age, marital status, place of residence, education, employment status and household income.

Smoking continuation showed a different pattern as regards its association with education and income. Among men, the association with smoking continuation remained statistically significant for low income but not for education in the multivariate model. Men in the lowest income group were about two times more likely to continue smoking when compared to the highest income group and a similar association (OR = 1.8) was found for women in the lowest income group. Unlike for men, low educational level was associated with smoking continuation among women. Women with primary education or less had more than two times higher odds to continue smoking compared to the highest educated. Employment status was not associated with smoking continuation among either men or women.

The divorced, separated or widowed men and women were more likely to have initiated smoking and to continue smoking (men only) when compared to those being married. Also, the never married men and women had higher odds for smoking continuation. Rural women were less likely to have initiated smoking when compared to urban women, but no differences were observed for smoking continuation. No urban/rural differences were observed among men for either of these measures.

**Figure 1:**
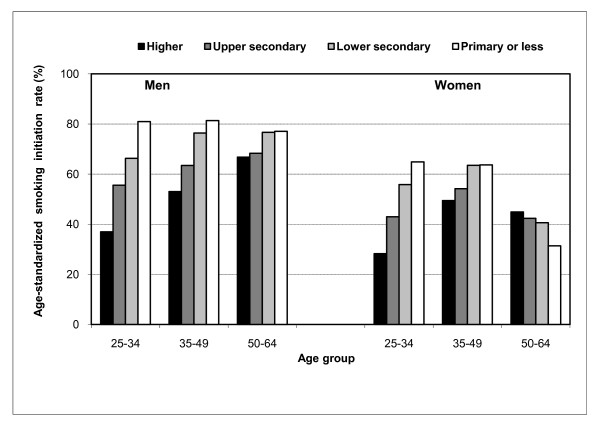
**Age-standardized rate (%) for smoking initiation by educational level in three age groups in Hungary, 2000-2003**.

**Figure 2:**
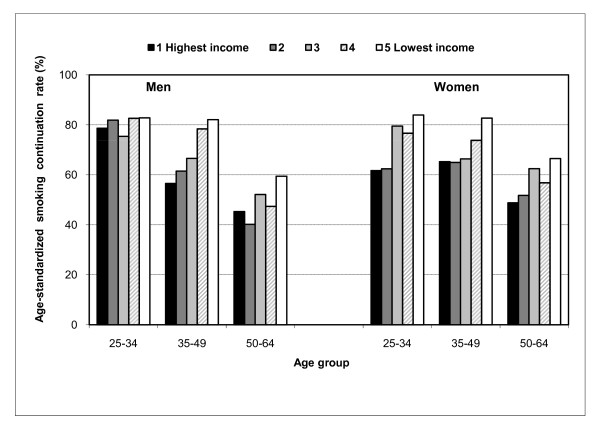
**Age-standardized rate (%) for smoking continuation by household income level in three age groups in Hungary, 2000-2003**.

Socioeconomic differences for smoking initiation and continuation in three age groups are presented in Table 3. The negative social gradient, when found, was almost always stronger in the youngest age group. For example, in the 25-34 age group, 81% of men with primary education or less had ever initiated smoking compared with only 37% of men with higher education; the corresponding figures for women were 65% and 28% (see Figure 1). At the same time, in the 50-64 age group, women with higher education were more likely to have initiated smoking, though the association was statistically not significant. An exceptional age pattern was observed for smoking continuation by income, for which the largest differences were found for middle-aged men (Figure 2). Though we did not observe statistically significant differences between manual and non-manual occupations as regards smoking initiation or continuation in any age group, we found nearly three times higher odds for smoking continuation among 25-34 years old men who were not employed when compared to men in non-manual occupations (Table 3). An association in the opposite direction was found for non-employed women in the same age group.

**Table 3:** Odds ratios for smoking initiation and continuation in three age groups in Hungary, 2000-2003.

		Odds ratios (with p-values)											
		
		Men						Women					
			
		Smoking initiation			Smoking continuation			Smoking initiation			Smoking continuation		
					
		25-34	35-49	50-64	25-34	35-49	50-64	25-34	35-49	50-64	25-34	35-49	50-64
													
**Education**													
	Higher	1.00	1.00	1.00	1.00	1.00	1.00	1.00	1.00	1.00	1.00	1.00	1.00
	Upper secondary	2.33**	1.62*	1.07	0.76	1.28	1.02	1.89**	1.16	1.04	1.37	1.31	0.98
	Lower secondary	3.55***	2.71***	1.31	0.56	1.54	1.23	2.58**	1.70*	0.94	3.25**	2.26*	0.75
	Primary or less	6.90***	3.35***	1.07	0.76	1.96	1.16	4.44***	1.52	0.67	7.11**	2.88**	1.13
													
**Employment**													
	Non-manual employees	1.00	1.00	1.00	1.00	1.00	1.00	1.00	1.00	1.00	1.00	1.00	1.00
	Manual workers/farmers	0.83	1.27	1.27	1.98	0.72	0.83	1.19	1.16	1.08	0.79	1.23	1.65
	Self-employed	0.93	1.05	0.64	0.96	0.60	1.20	2.64*	1.17	0.53	1.41	1.21	0.34
	Non-employed	0.75	1.17	1.56	2.89**	0.64	0.76	0.90	1.38	1.01	0.30**	0.94	1.30
													
**Household income**													
	1 Highest	1.00	1.00	1.00	1.00	1.00	1.00	1.00	1.00	1.00	1.00	1.00	1.00
	2	1.23	0.78	0.77	1.12	1.21	0.83	1.24	0.68*	0.94	1.03	0.84	0.85
	3	0.94	1.08	0.88	0.72	1.41	1.39	1.05	0.91	1.03	2.10	0.77	1.26
	4	1.15	1.05	0.80	1.11	2.48**	1.05	1.58	0.80	0.90	1.87	0.96	0.88
	5 Lowest	1.34	1.14	1.09	0.93	3.04***	1.74	3.01***	1.18	1.13	2.45**	1.66	1.39

All estimates were adjusted for age, marital status, place of residence, education, employment status and household income.

*** (p < 0.001); ** (p < 0.01); * (p < 0.05)

Since the effect of educational level on smoking initiation varied considerably by age group, we tested if there was a notable interaction between age and education in the association with smoking initiation. The interaction term was statistically significant, indicating that age is an important effect modifier in the association between education and smoking initiation.

## Discussion

### Summary of findings

Unlike in the more developed countries to the West, in Hungary, education and income (especially the lowest level), but not employment, were associated with equally large differences in smoking prevalence. Among men, the effect of education on smoking prevalence was determined mostly through its impact on smoking initiation, whereas the effect of income was more important for smoking continuation. Among women, income and education had an impact on both smoking initiation and continuation. The negative association between educational level and smoking initiation was strongest in the youngest group of men and women, whereas for women in the oldest age group this association was positive, though not statistically significant. The effect of income on smoking continuation was particularly strong among middle-aged men.

### Potential limitations

The overall non-response rate in the two surveys was 21 and 27%. We do not have data about the non-respondents' socioeconomic status or smoking behaviour. Non-response tends to be more common among lower socioeconomic groups and among smokers and may therefore affect the overall prevalence rate, however, it is unlikely to affect the associations when socioeconomic inequalities are studied [22-24].

The item non-response was high mainly for household income (about 14%). Men who did not report their income, tended to be slightly higher educated and to smoke more, whereas among women no such differences were observed. The larger income non-response among higher socioeconomic groups has also been reported by other studies [25]. Assuming that the higher educated also have a higher income, we may have missed more smokers in the high income group and subsequently overestimated the association between income and smoking. On the other hand, for those who do report their income, there is the possibility of a general tendency to underreport income. This is believed to be a common practice in Central and Eastern Europe because of the larger share of income from unofficial sources [26]. If it has any effect at all, the underreporting of income by some of the respondents, may have led to an underestimation of the association between income and smoking, thus balancing the effect of income non-response.

Because of the very high levels of smoking attributable mortality in Hungary we cannot exclude the possible mortality selection bias in our study. This affects our estimates only if the relative risk of mortality due to the exposure to smoking is higher in lower socioeconomic groups than in higher socioeconomic groups. In that case we may have underestimated the association between socioeconomic status and smoking, although it is unlikely that the effect would be very different for education compared to income.

We did not observe any association between non-manual and manual occupations in the multivariate analysis, which can be related to our less discriminative measure of employment when compared to other studies [12,15]. For example, we were not able to distinguish between the higher and lower non-manual occupations, although we found important differences in smoking behaviour between these two groups in our earlier study from Estonia [12]. However, when compared to non-manual employees, a statistically significant association with smoking continuation was observed in the youngest age group among the non-employed.

Finally we also have to note that the cross-sectional design of the study does not allow causal associations to be established, but rather enables the patterns of inequalities linked in some way to different socioeconomic indicators to be revealed.

### Interpretation of the results for education

Educational level was negatively related to smoking initiation among both men and women. The only exception was the positive relationship between smoking initiation and educational level among women in the age group 50-64 (i.e. those born in 1936-1953). This pattern is fully consistent with what could be expected according to the smoking epidemic model, formulated on the basis of the experience of more developed countries [7]. In the northern European countries, a positive association between smoking and educational level has disappeared even among women born in 1950 or earlier [13]. The persistence of the positive relationship among Hungarian women in the same birth cohort indicates that Hungary is lagging behind by at least 15 years. However, when compared to most of the former Soviet countries [8-10], the reversal of social gradient in smoking occurred earlier among Hungarian women. The country's closer proximity to Western countries and its more open economy could be two contributory factors that accelerated the earlier uptake of smoking among Hungarian women. Ultimately, this may be related to both the influx of ideas concerning modernization and women's emancipation [13] but also to their better access to western brands of cigarettes.

The largest educational differences in ever initiating smoking were found in the 25-34 age group, i.e. the age group that initiated smoking largely in the 1990 s. The widening educational gap in smoking initiation across generations is mostly driven by the declining initiation rate among the highest educated. At the same time no decline was observed among the lowest educated which may partly reflect the consequences of the tobacco industry's expansion. In the early 1990 s, exploiting the legislative loopholes in the immediate post-transition period, transnational tobacco companies rapidly expanded their markets into Eastern Europe [27]. High overall consumption levels, a rising smoking prevalence among women, and the country's geographical location between the West and East, made the Hungarian market particularly attractive [28].

Smoking initiation usually relates to the period of adolescence, which may explain the importance of the individual's educational level for smoking initiation compared to the indicators of adult socioeconomic position. In the youngest cohorts, prolonged education may directly prevent the early uptake of smoking via better knowledge about potential health hazards [29], or indirectly via personality traits like higher self-esteem and internal locus of control [30,31]. In addition, early school dropout may be related to life-course trajectories that are also associated with early smoking uptake [32]. Those who come from lower class families are more likely to have been exposed to adverse experiences [33] and adverse living conditions in childhood [34]. Dropping out of school may also lead to an early start as regards an individual's working life and as a result of this, to the availability of disposable income, thus making cigarettes more accessible. On the other hand, income differences have been considered less important in lower class adolescents because of their better access to tobacco from family and friends, and to cheaper cigarettes from illegal sources [35]. According to more conservative estimates, the black market accounts for at least 5-10% of the Hungarian cigarette market [36].

For smoking continuation, the independent effect of education was found only among women, whereas among men, the effect of education was explained by income differences. The stronger effect of education among women may be related to their greater propensity to make knowledge-based decisions as regards health behaviours [31]. Higher educated women are also more likely to stop smoking while becoming pregnant [37,38]. The fact that child bearing and motherhood in general are likely to be associated with smoking cessation among women is supported by the finding that non-employed women in the youngest age group had the lowest continuation rates when compared to those in employment. This was also in sharp contrast with non-employed men in the same age group who had the highest risk of continuing smoking.

### Interpretation of the results for income

Income was associated with smoking prevalence as strongly as educational level in this study. In this respect, income is a more important predictor of smoking in Hungary than in economically more developed European countries. A stronger association with material disadvantage, compared to educational level, was also found for smoking prevalence in Russia [39]. Similarly to our study, low income was found to be strongly related to high continuation rates in other economically less developed countries, especially among men [12,40].

The relationship between income and smoking prevalence was J-shaped, with by far the highest prevalence rates among men and women in the lowest income quintile. This income group more likely represents people living in severe poverty. It has been argued that poverty may underpin the increasing social inequalities in mortality in Eastern Europe [41], and poverty-related psychosocial stress might also explain part of the increased risk of smoking among the poorest. The fact that stressful life events may strongly influence smoking behaviour is illustrated by our finding that divorced or widowed persons had a higher smoking prevalence compared to married individuals. Similarly, the finding that young men who were not employed had high continuation rates even after controlling for the effect of low income also adds some support to stress related explanations.

Low income was particularly related to high smoking continuation rates, especially among men. Faced with financial stress, smokers may be less likely to quit, while ex-smokers may be more likely to relapse [42]. Smoking has been considered as an affordable palliative for coping with stress among the poor [43]. Smokers from lower socioeconomic groups also have a higher smoke intake and may thus be more nicotine dependent [44], making smoking cessation more difficult. In addition, effective smoking cessation services may be more expensive and hence less affordable for the poor.

In Hungary, there may be high smoking rates among the poor partly due to low cigarette prices. By using active lobbying strategies, the transnational tobacco companies were able to prevent substantial price increases being made on cigarettes until the early 2000 s [45,46]. In 2001, the cigarette price in Hungary was still among the lowest in Europe. Compared to the UK, the price of local brand cigarettes was eight times lower in terms of $US, and still three times lower in terms of domestic affordability [47]. We should add however, that the effect of price on tobacco consumption, especially among those in the lower socioeconomic groups is as yet uncertain [48]. Price increases may be less effective among the poor, as low income smokers are more likely to purchase smuggled or home-made cigarettes [49]. Besides, when prices are high already, further increasing tobacco taxation may impose a disproportionate burden on smokers with already low incomes [50].

## Conclusions

As regards to our study hypothesis it can be concluded that income matters very much to tobacco consumption in a country like Hungary, most likely because of the combination of poverty among the lowest income groups, and the affordability of cigarettes. At the same time, education maintains its independent effect on smoking, which seems to be developing in Hungary in precisely the same way it did in the more affluent countries. Inequalities in smoking in economically less developed countries can thus be best understood in relation to two processes: the smoking epidemic, and the effects of poverty.

If no special efforts are made to curtail the smoking epidemic, inequalities in smoking are likely to widen in Hungary. The affordability of cigarettes decreased by 20% from 2003 to 2004 in conjunction with the Hungarian government's efforts to adapt to EU tax levels, and as a result, the daily consumption of cigarettes per smoker decreased [36]. In order to reduce tobacco consumption among the lower socioeconomic groups, a tax increase alone would be insufficient if no attempt is made to control the trade in contraband cigarettes. In addition, this has to be accompanied by other tobacco control measures, for example, removing financial barriers to nicotine replacement therapy and other smoking cessation services, and by implementing school-based smoking prevention programmes already at lower educational levels [51].

## Competing interests

The authors declare that they have no competing interests.

## Authors' contributions

ML outlined the paper, did the data analysis, discussed core ideas, drafted the paper and prepared the final manuscript. CK prepared the dataset and commented on drafts. AEK led the project, discussed core ideas and commented extensively on drafts. All authors read and approved the final manuscript.

## Pre-publication history

The pre-publication history for this paper can be accessed here:

http://www.biomedcentral.com/1471-2458/11/97/prepub
